# Supplementary material for: Bridging scales in disordered porous media by mapping molecular dynamics onto intermittent Brownian motion
Source: Nat Commun. 2021 Feb 15;12:1043. doi: 10.1038/s41467-021-21252-x (PMC7884405; doi:10.1038/s41467-021-21252-x)
Supplement: Supplementary file 1 — Supplementary Information [file 41467_2021_21252_MOESM1_ESM.pdf]

Supplementary Information: Bridging scales in disordered porous media by mapping molecular dynamics onto Intermittent Brownian Motion

Colin Bousige,[1,*] Pierre Levitz,[2,†] and Benoit Coasne[3,‡]

[1] *Univ. Lyon, Université Claude Bernard Lyon 1, CNRS UMR 5615, Laboratoire des Multimatériaux et Interfaces, F-69622 Villeurbanne, France*

[2] *Sorbonne Université, CNRS UMR 8234, PHENIX Lab, 75252 Paris, France*

[3] *Univ. Grenoble Alpes, CNRS, LIPhy, 38000 Grenoble, France*

(Dated: January 13, 2021)

Contents

I. Supplementary Notes 2

Supplementary Note 1. Relocation time and the formalism of mean first passage times 2

Supplementary Note 2. Data treatment and fits 4

II. Supplementary Figures 5

III. Supplementary Tables 12

IV. Supplementary References 12

*Electronic address: colin.bousige@univ-lyon1.fr

†Electronic address: pierre.levitz@sorbonne-universite.fr

‡Electronic address: benoit.coasne@univ-grenoble-alpes.fr

I. SUPPLEMENTARY NOTES

Supplementary Note 1. Relocation time and the formalism of mean first passage times

Relocation on a semi-infinite medium. As shown in Ref. [1], ψ_B^∞ can be determined by considering the trajectory of a molecule starting at a distance x_0 from the adsorbing planar region located in $x = 0$ and crossing for the first time this interface at a time t. In practice, assuming Fickian diffusion upon relocation in this semi-infinite medium, $\psi_B^\infty(t)$ can be estimated from the particle distribution function $c(x,t)$ by considering the flux of molecules reaching the interface $x = 0$ at a time t:

$$\psi_B^\infty(t) = +D_s^p \frac{\partial c(x,t)}{\partial x}\bigg|_{x=0} \text{ with } t \to +\infty \tag{1}$$

Such an equation is reminiscent of Fick's first law with a sign '+' to account for the fact that we are counting molecules entering (not leaving) the adsorption domain. Within the Fickian diffusion assumption, $c(x,t)$ can be expressed as:

$$c(x,t) = \frac{1}{\sqrt{4\pi D_s^p t}} \times \left[\exp\left(-\frac{(x-x_0)^2}{4D_s^p t}\right) - \exp\left(-\frac{(x+x_0)^2}{4D_s^p t}\right)\right] \tag{2}$$

This equation differs from the conventional Gaussian distribution by the second exponential term which corresponds to an anti-Gaussian distribution ensuring that the adsorbing domain is an absorbing point for the relocating molecules, *i.e.* $c \sim 0$ in this region (this method is known as the image method in statistical physics). By noting that $c(x,t) \sim [4\pi(D_s^p t)^3]^{-1/2} \times x x_0 \exp[-(x^2 + x_0^2)/4D_s^p t]$, Eq. (S1) leads to the following solution:

$$\psi_B^\infty(t) = \frac{x_0}{\sqrt{4\pi D_s^p t^3}} \exp\left(-\frac{x_0^2}{4D_s^p t}\right) \underset{t\to+\infty}{\sim} \frac{x_0}{\sqrt{4\pi D_s^p t^3}} \tag{3}$$

where the second equality corresponds to the solution in the long time limit in the case of diffusion in the semi-infinite space above a planar surface.

Relocation in a finite, *i.e.* confining, medium. In a confining pore network, the long time evolution of $\psi_B(t)$ is sensitive to the finite pore size. As discussed elsewhere [2], $\psi_B(t)$ exhibits an exponential tail at long time as found numerically in the inset of Fig. 3. $\psi_B(t)$ can therefore be written as $\psi_B(t) = \psi_B^\infty(t)\exp(-t/t_c) = C\exp(-t/t_c)/t^{3/2}$ for $t > t_0$. On the one hand, the short time cutoff t_0 is the time of the minimal bridge having a spacial extension $x_{\min}$. On the other hand,

t_c is associated to a geometrical cut-off length r_c which gives the maximal extension of a bridge. r_c, which is of the order of the pore size p, can be written as $r_c = \beta p$ where $\beta \sim 1$ is related to the accessible in-pore horizon. Computing the normalization condition, $\int_{t_0}^{\infty} C \exp(-t/t_c)/t^{3/2} \mathrm{d}t = 1$, we get the following expression for the constant C:

$$\begin{aligned}\frac{1}{C} &= \int_{t_0}^{\infty} \frac{e^{-t/t_c}}{t^{3/2}} \mathrm{d}t = \left[-2\frac{e^{-t/t_c}}{\sqrt{t}} \right]_{t_0}^{\infty} - \int_{t_0}^{\infty} -2\frac{e^{-t/t_c}}{t_c\sqrt{t}} \mathrm{d}t \\ &= \frac{2e^{-t_0/t_c}}{\sqrt{t_0}} - \frac{2}{t_c} \left(\underbrace{\int_0^{\infty} \frac{e^{-t/t_c}}{\sqrt{t}} \mathrm{d}t}_{I_1} - \underbrace{\int_0^{t_0} \frac{e^{-t/t_c}}{\sqrt{t}} \mathrm{d}t}_{I_2} \right)\end{aligned} \tag{4}$$

where the second equality corresponds to an integration by parts. Introducing in I_1 and I_2 the variable change $u^2 = t/t_c$ so that $\mathrm{d}t/\sqrt{t} = 2\mathrm{d}u\sqrt{t_c}$, we get:

$$\begin{aligned} I_1 &= 2\sqrt{t_c} \int_0^{\infty} e^{-u^2} \mathrm{d}u = \sqrt{\pi t_c} \\ I_2 &= 2\sqrt{t_c} \int_0^{\sqrt{t_0/t_c}} e^{-u^2} \mathrm{d}u = \sqrt{\pi t_c}\, \mathrm{erf}(\sqrt{t_0/t_c}) \\ I_1 - I_2 &= \sqrt{\pi t_c}\, \mathrm{erfc}\left(\sqrt{\frac{t_0}{t_c}} \right) \end{aligned}$$

where $\mathrm{erfc}(x) = 1 - \mathrm{erf}(x)$ is the complementary error function. Injecting this last expression into Eq. (4) leads to:

$$C = 1 \Bigg/ \left[\frac{2e^{-t_0/t_c}}{\sqrt{t_0}} - 2\sqrt{\frac{\pi}{t_c}}\, \mathrm{erfc}\left(\sqrt{\frac{t_0}{t_c}} \right) \right] \tag{5}$$

Then, the first moment t_B of $\psi_B(t)$ can be obtained by assuming that $t_0 \ll t_c$ and developing the erfc function to the second order:

$$\begin{aligned} t_B &= \int_{t_0}^{\infty} t\psi_B(t) \mathrm{d}t \\ &= C(I_2 - I_1) \\ &= \sqrt{\pi t_c}\, \mathrm{erfc}\left(\sqrt{\frac{t_0}{t_c}} \right) \Bigg/ \left[\frac{2e^{-t_0/t_c}}{\sqrt{t_0}} - 2\sqrt{\frac{\pi}{t_c}}\, \mathrm{erfc}\left(\sqrt{\frac{t_0}{t_c}} \right) \right] \\ &\underset{t_0 \ll t_c}{\simeq} \sqrt{\pi t_c} \left(1 - \frac{2}{\sqrt{\pi}} \sqrt{\frac{t_0}{t_c}} \right) \Bigg/ \left[\frac{2}{\sqrt{t_0}} - 2\sqrt{\frac{\pi}{t_c}} \right] \\ &\underset{t_0 \ll t_c}{\simeq} \frac{\sqrt{\pi t_c t_0}}{2} - t_0 \end{aligned} \tag{6}$$

On the one hand, t_c is associated with a geometrical cut-off length r_c corresponding to the maximal bridge extension. r_c, which is of the order of the pore size d, writes $r_c = \beta d$ with $\beta \sim 1$. On the other hand, t_0 is related to the short-range threshold time for the scaling $t^{1/2}$ associated to the typical distance $x_{\min}$. Assuming Fickian diffusion upon relocation, we can write $t_0 \sim x_{\min}^2/2D_s^p$ and $t_c \sim \beta^2 d^2/2D_s^p$. As discussed in the main text, a simple estimation able to capture the overall data behavior in Fig. 4d (obtained for $\varepsilon/k_{\mathrm{B}}T = 1$) with a scaling $t_B(d) \sim d$ and a negative intercept $t_B(0) < 0$ leads to $x_{\min} \sim 0.12$ nm and $\beta \sim 0.4$. With this two-parameter fit, $x_{\min}$ is considered independent of the pore structure. Yet, a more advanced analysis can be performed by inspecting the probability density function (PDF) of the bridge displacement $\theta(r)$ with r the end-to-end Euclidean of a Brownian bridge [3] [see Fig. 10(a)]. As shown in Fig. 10(a), the bridge displacement follows a power law, *i.e.* $\theta(r) \sim r^{-2}$, in the intermediate range which is typical of flat surfaces. While an exponential cutoff is observed at large distances, a maximum is observed, associated with the end of the algebraic regime at short distances. Using this maximum to define $x_{\min}$, Fig. 10(b) shows that $x_{\min}$ depends on pore diameter d with a nearly linear increase for d varying from 0.02 to 0.05 nm. As shown in Fig. 10(c), taking into account this evolution, the generic behavior for $t_B \times D_s^p$ as a function of d can be retrieved with a unique value $\beta = 0.7$ (for all $\varepsilon/k_{\mathrm{B}}T$).

Supplementary Note 2. Data treatment and fits

All data treatment, fits and figures were made using the open source language R [4]. The fit for $D_s^p(d)$ in the inset of Fig. 2(a) was performed by applying Eq. (2) to the simulated data using the nls() function in R. The nls() function performs Non-Linear Least Squares fitting using the Gauss-Newton algorithm. The initial guesses used are $D_0 = 12 \times 10^{-9}$ m^2/s, $D_s^s = min[D_s]$, $\sigma = 2$ Å, and $r_0 = 1$ Å. The error bars on Supplementary Fig. 7 were obtained from the fit by leaving all four parameters free (the standard errors on the fitting parameters are output by the nls() function). To assess the fit quality, several fits were performed by imposing the values of D_0 or σ; we found that the fit parameters converge to the same values as those shown in Supplementary Fig. 7. In the end, we chose to show Fig. 2(a) and Supplementary Fig. 7 with four free parameters to show that the impact of $\varepsilon/k_B T$ of these parameters does not depend on our choice to impose a given parameter or another.

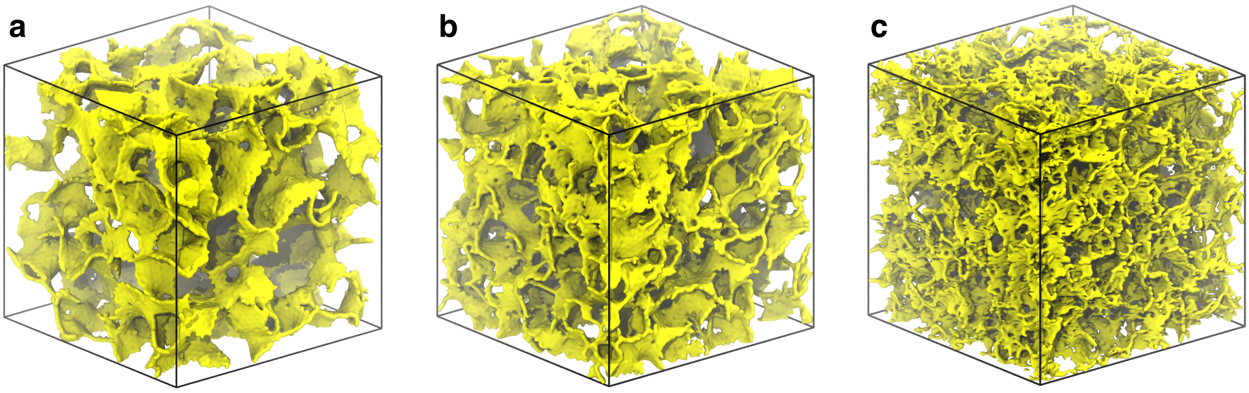

Supplementary Figure 1: Molecular configurations of the porous materials $CS_{0.5}$ (a), $CS_{0.75}$ (b) and $CS_{1.0}$ (c). The corresponding skeleton densities are 0.5 g/cm^3, 0.75 g/cm^3 and 1.0 g/cm^3.

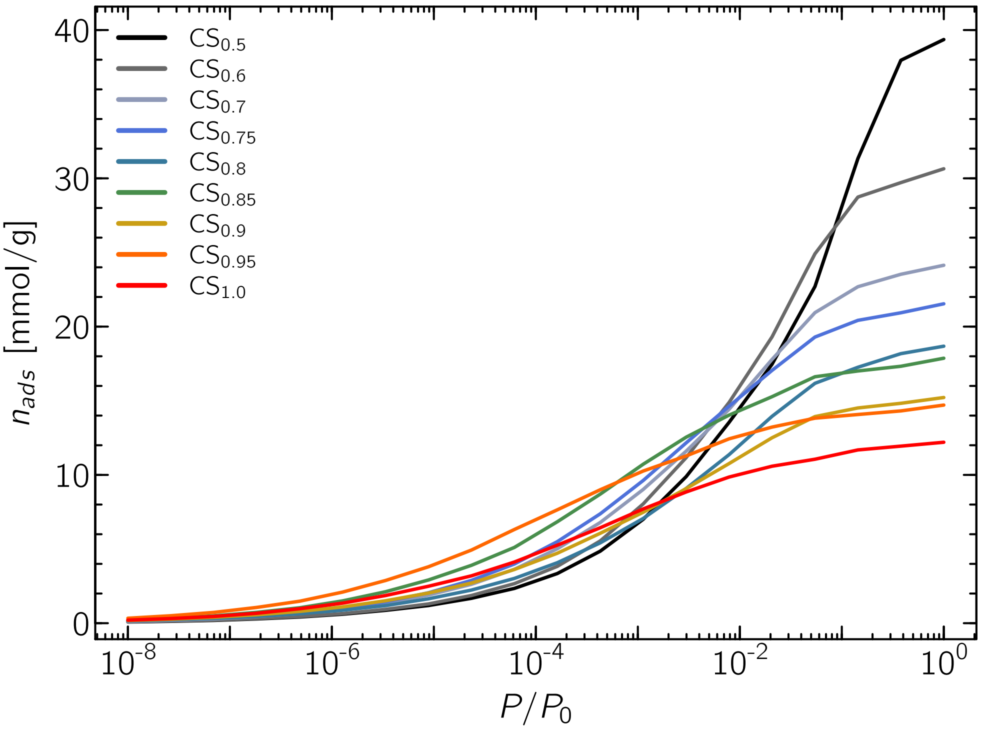

Supplementary Figure 2: Methane adsorption isotherms at 111.7 K for the 9 different samples listed in bold in Tab. 1. These data were obtained using the GCMC algorithm as described in the *Methods* section in the main text. The pressure axis is normalized to the bulk saturating vapor pressure, $P_0 = 101325$ Pa.

II. SUPPLEMENTARY FIGURES

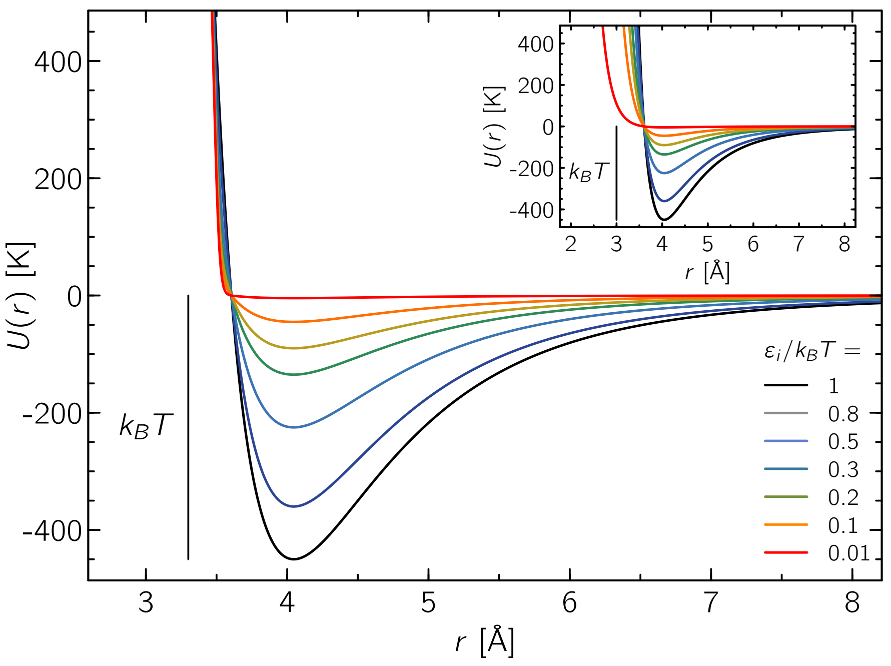

Supplementary Figure 3: Modified Lennard-Jones interaction potential as defined using Eqs. (11-12) for different parameters ε_+ (indicated in the graph). This simple interaction potential allows keeping the repulsive contribution constant while varying the the fluid/surface interaction strength. The inset shows the standard 12-6 Lennard-Jones potential with varying the well-depth ε_+.

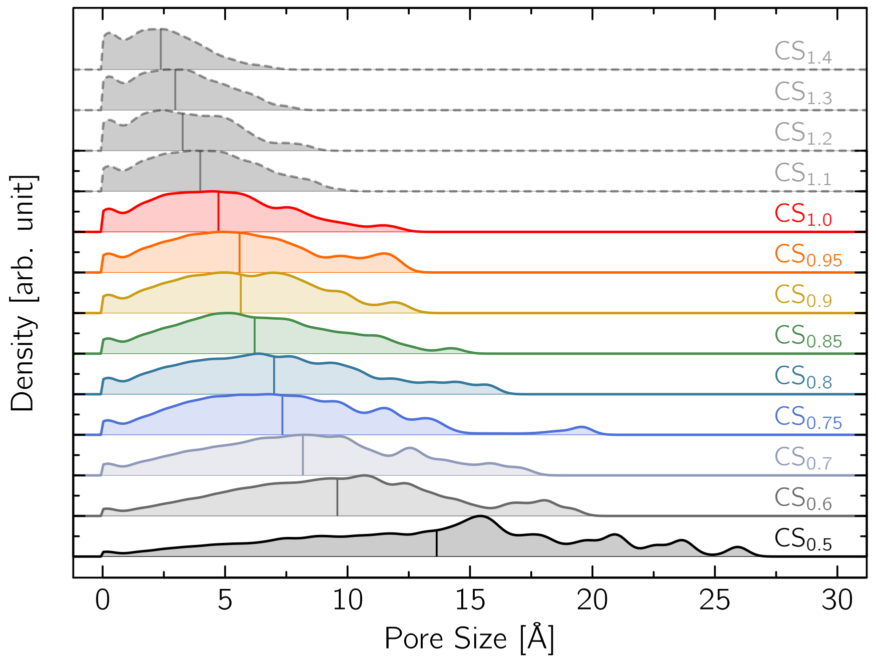

Supplementary Figure 4: Pore Size Distribution for the 13 different nanoporous solids listed in Tab. 1. These data were obtained using the simple stochastic algorithm as described in the main text. For each sample, the vertical line indicate the corresponding mean pore size d.

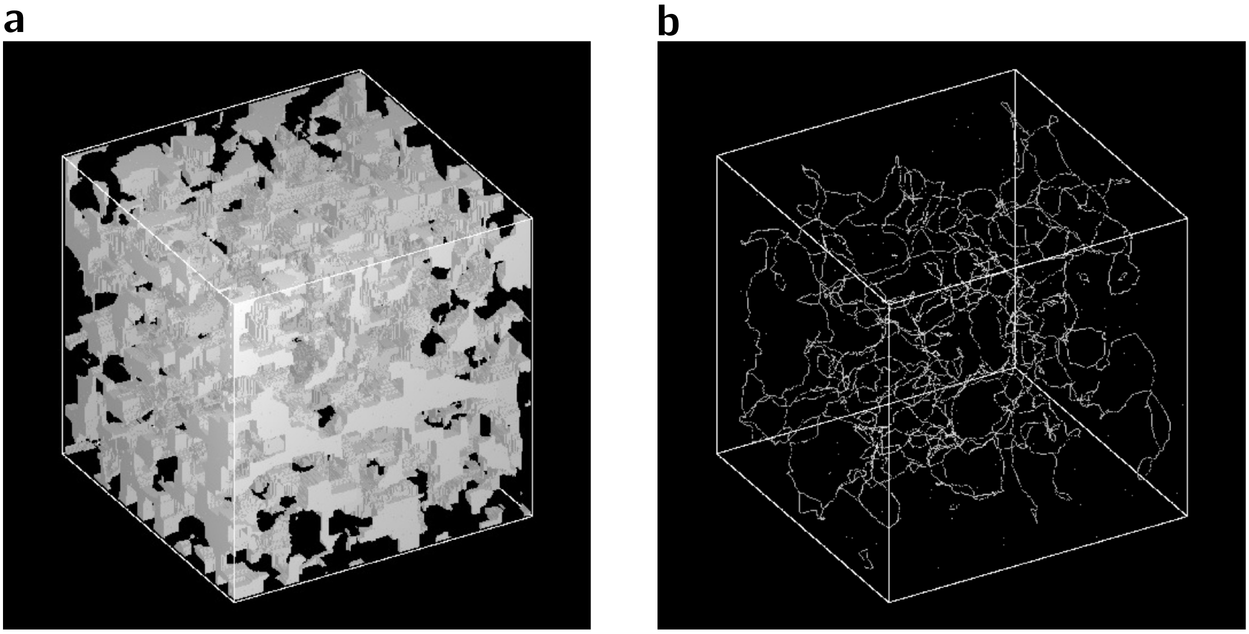

Supplementary Figure 5: (a) Digitized pore network corresponding to the nanoporous solid $CS_{1.0}$. (b) Retraction graph for the sample shown in (a) with a porosity $\phi = 0.18$. The voxel size is 0.2 Å and the box size 100 Å.

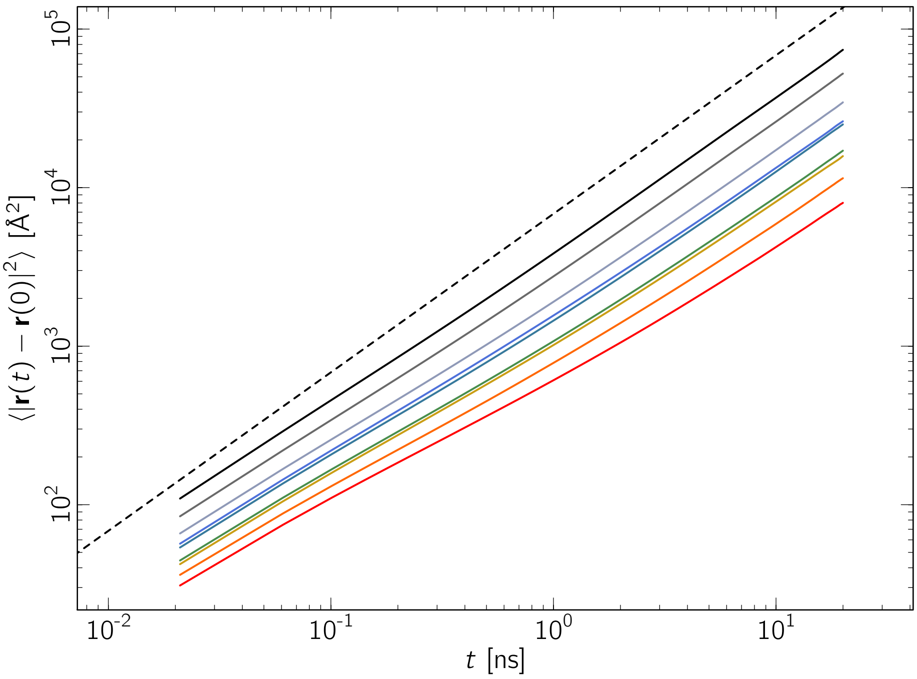

Supplementary Figure 6: Mean square displacements for the different nanoporous carbon structures and $\varepsilon/k_\mathrm{B}T = 0.1$. The dashed line corresponds to the data for bulk methane from which the bulk self-diffusivity D_s^0 can be derived.

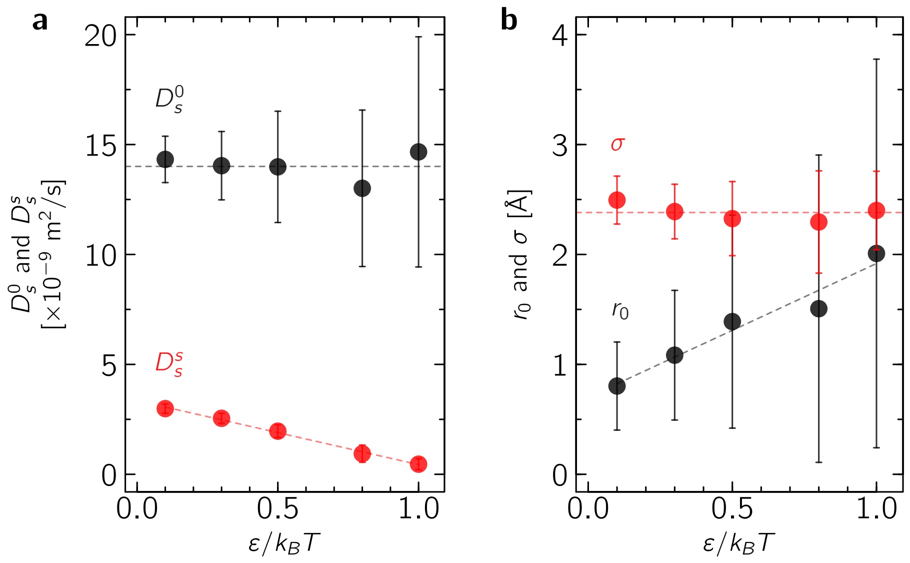

Supplementary Figure 7: Parameters of the effective fit shown in the inset of Fig. 3 in the main text as a function of $\varepsilon/k_{\rm B}T$: (a) D_s^s and D_s^0, and (b) σ and r_0. While D_s^s and r_0 evolve linearly with $\varepsilon/k_{\rm B}T$, $D_s^0 = 14 \pm 0.6 \times 10^{-9}$ m^2/s and $\sigma = 2.4 \pm 0.1$ Å are found to be constant. The vertical bars correspond to fitting procedure errors.

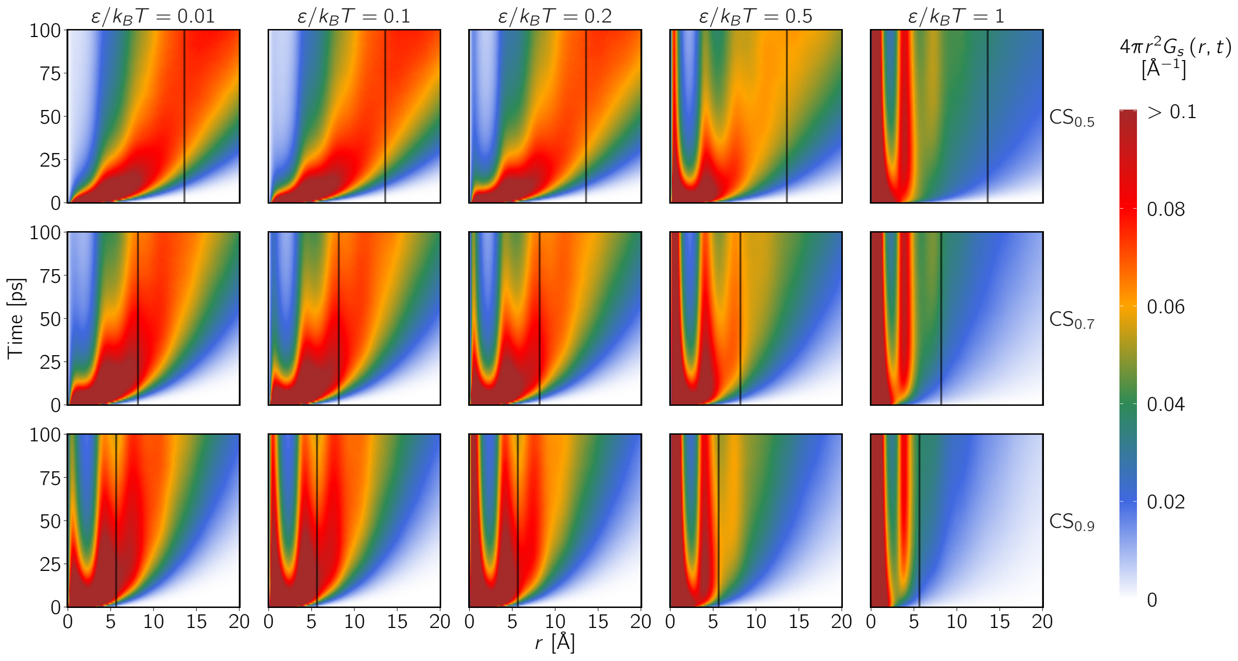

Supplementary Figure 8: Contour plot showing the self-correlation function $4\pi r^2 \times G_s(r,t)$ for methane in the disordered nanoporous solids $CS_{0.5}$, $CS_{0.7}$ and $CS_{0.9}$. For each sample, the data are shown for various ε/k_BT in a given column. For each system, the vertical lines indicates the the mean pore size d.

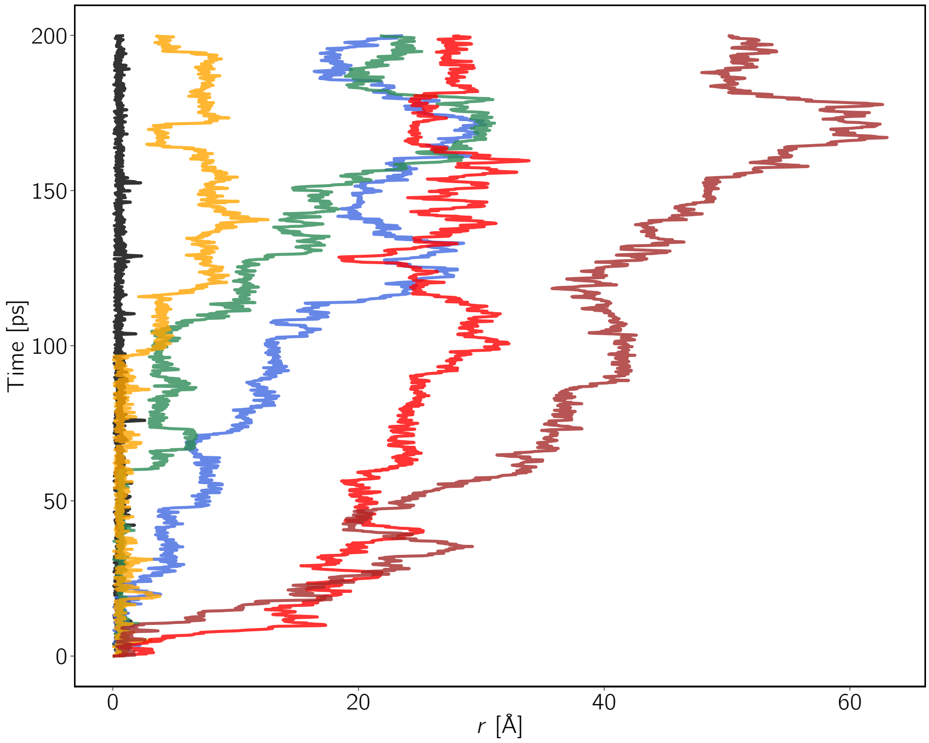

Supplementary Figure 9: Time evolution of the displacement vector $r(t)$ for 6 random molecules in the structure $CS_{0.7}$ for $\varepsilon/k_BT = 0.5$. Some molecules are stuck in a small pore (black) while other molecules diffuse throughout the structure (brown). Most molecules show a behavior in between these two extrema with well-defined relocation and adsorption periods.

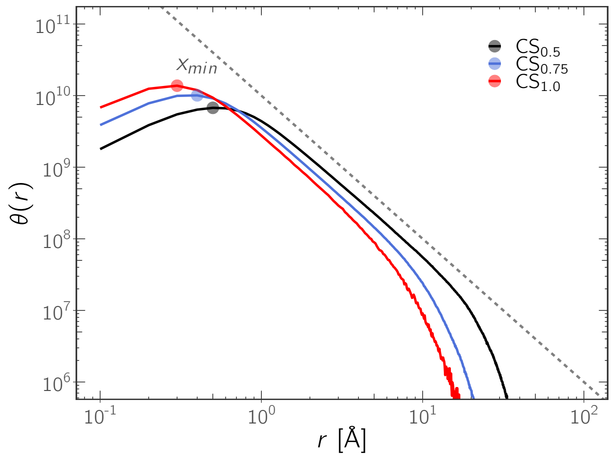

(a)

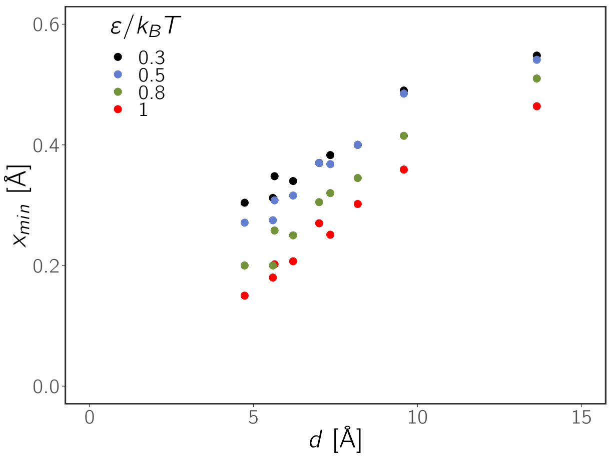

(b)

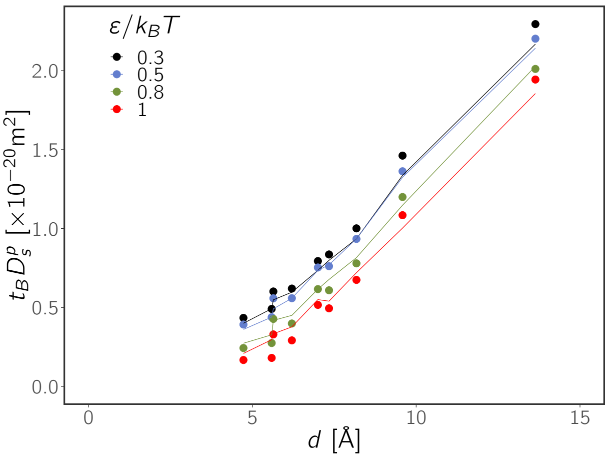

(c)

Supplementary Figure 10: (a) Evolution of the probability density function of the bridge displacement $\theta(r)$, with r the end-to-end Euclidean of a Brownian bridge. Computation was performed for $\varepsilon/k_\mathrm{B}T = 0.5$. The grey dashed line follows an algebraic law r^{-2}. The large points mark the positions of x_{min} for the various samples. – (b) Evolution of x_min with the pore diameter p, for different $\varepsilon/k_\mathrm{B}T$ ratios. – (c) $t_B \times D_s^p$ versus the mean pore size d for different $\varepsilon/k_\mathrm{B}T$ ratios. Comparison between numerical simulations (points) and predictions with Eq. (8) of the main text with an unique value $\beta = 0.7$ (lines).

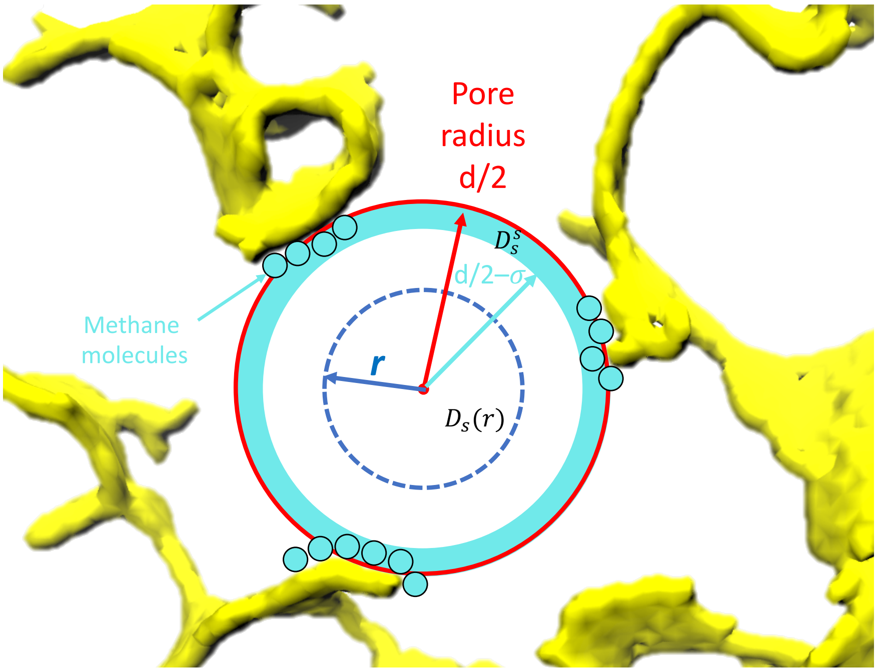

Supplementary Figure 11: Schematic representation showing the simple effective model based on the local self-diffusivity $D_s(r)$ given in Eq. (2). For $r > d/2-\sigma$, molecules are adsorbed at the surface with a constant self-diffusivity $D_s(r) = D_s^s$. For $r < d/2 - \sigma$, the molecules are located in the pore center with a local self-diffusivity $D_s(r)$ depending on r. In this approach, $r = 0$ is the pore center where the diffusivity tends to the bulk value D_s^0 provided the pores are large enough.

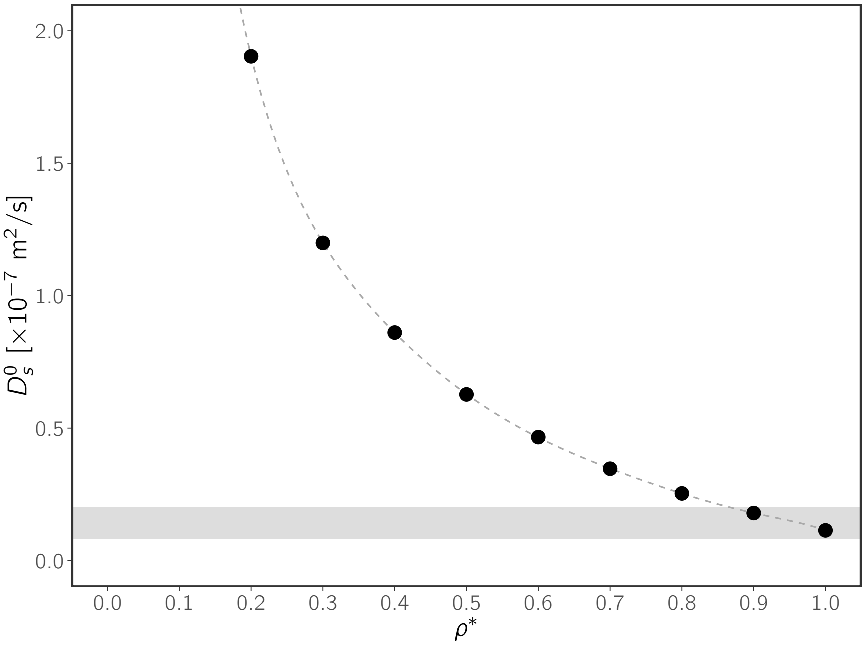

Supplementary Figure 12: Self-diffusivity D_s^0 of bulk methane at $T = 450$ K as a function of the reduced density $\rho^* = N/\sigma^3/V$. The dashed line is a guide to the eye while the shaded area indicates $D_s^0 = 14 \pm \times 10^{-9}$ m^2/s as found in Fig. 7.

III. SUPPLEMENTARY TABLES

Supplementary Table 1: Density ρ_s, porosity ϕ, mean pore size d, and connection number c_t for the different disordered nanoporous samples considered CS_{XX}. Only the porous solids marked in bold were considered to investigate fluid transport as the other samples possess a non-connected porous network ($c_t < 0$).

Sample	ρ_s [g/cm^3]	ϕ	c_t	d [Å]
$\mathbf{CS}_{0.5}$	0.5	0.52	0.43	13.6
$\mathbf{CS}_{0.6}$	0.6	0.43	0.45	9.58
$\mathbf{CS}_{0.7}$	0.7	0.35	0.39	8.18
$\mathbf{CS}_{0.75}$	0.75	0.31	0.34	7.34
$\mathbf{CS}_{0.8}$	0.8	0.28	0.36	7.00
$\mathbf{CS}_{0.85}$	0.85	0.26	0.26	6.21
$\mathbf{CS}_{0.9}$	0.9	0.23	0.22	5.64
$\mathbf{CS}_{0.95}$	0.95	0.22	0.24	5.59
$\mathbf{CS}_{1.0}$	1.0	0.18	0.16	4.73
$\mathrm{CS}_{1.1}$	1.1	0.14	-0.17	3.99
$\mathrm{CS}_{1.2}$	1.2	0.10	-0.36	3.27
$\mathrm{CS}_{1.3}$	1.3	0.07	-0.46	2.97
$\mathrm{CS}_{1.4}$	1.4	0.05	-0.61	2.38

Supplementary Table 2: Like-species Lennard-Jones parameters used for carbon, hydrogen, and methane in the GCMC and MD simulations [5]. The unlike-species parameters were determined using the Lorentz-Berthelot rules: $\sigma_{xy} = 1/2\,(\sigma_{xx} + \sigma_{yy})$ and $\varepsilon_{xy} = \sqrt{\varepsilon_{xx}\varepsilon_{yy}}$.

	C	H	CH_4
σ_{xx} (Å)	3.40	2.42	3.81
ε_{xx} (K)	28.0	15.1	148.1

IV. SUPPLEMENTARY REFERENCES

[1] Redner, S. *A Guide to First-Passage Processes* (Cambridge University Press, 2001).

[2] Levitz, P. Probing interfacial dynamics of water in confined nanoporous systems by NMRD. *Mol. Phys.* **117**, 952–959 (2019).

[3] Levitz, P., Grebenkov, D. S., Zinsmeister, M., Kolwankar, K. M. & Sapoval, B. Brownian Flights over a Fractal Nest and First-Passage Statistics on Irregular Surfaces. *Phys. Rev. Lett.* **96**, 180601 (2006).

[4] Team, R. C. R: A Language and Environment for Statistical Computing (2017).

[5] Billemont, P., Coasne, B. & De Weireld, G. Adsorption of carbon dioxide, methane, and their mixtures in porous carbons: Effect of surface chemistry, water adsorption, and pore disorder. *Langmuir* **29**, 3328–3338 (2013).
